# In Silico Designed Gain-of-Function Variants of Complement C2 Support Cytocidal Activity of Anticancer Monoclonal Antibodies

**DOI:** 10.3390/cancers14051270

**Published:** 2022-03-01

**Authors:** Aleksandra Urban, Alan Majeranowski, Grzegorz Stasiłojć, Patrycja Koszałka, Anna Felberg, Michał Taszner, Jan M. Zaucha, Marcin Okrój

**Affiliations:** 1 Department of Cell Biology and Immunology, Intercollegiate Faculty of Biotechnology, University of Gdańsk and Medical University of Gdańsk, Dębinki 1 Street, 80-211 Gdańsk, Poland; aleksandra.urban@gumed.edu.pl (A.U.); alan.majeranowski@gumed.edu.pl (A.M.); gstasilojc@gumed.edu.pl (G.S.); pkosz@gumed.edu.pl (P.K.); anna.felberg@gumed.edu.pl (A.F.); 2 Department of Hematology and Transplantology, Medical University of Gdańsk, Smoluchowskiego 17 Street, 80-214 Gdańsk, Poland; mitaszner@gumed.edu.pl (M.T.); jan.zaucha@gumed.edu.pl (J.M.Z.)

**Keywords:** complement system, CLL, NHL, rituximab, obinutuzumab

## Abstract

**Simple Summary:**

The complement system can be exploited by anticancer antibody-based therapeutics. Activation of the classical complement pathway by the heavy chain of antibodies eventually leads to the lysis of target cells. However, overexpression of complement inhibitors by tumor cells limits the therapeutic efficacy. We designed and produced recombinant gain-of-function variants of complement C2 protein that counteract the activity of complement inhibitors. The dominant character of designed mutations in C2 allows supplementation of human serum with recombinant proteins for enhancing the cytocidal activity of complement-activating antibodies. In vitro functional assays demonstrate that this strategy is compatible with several clinically approved antibodies.

**Abstract:**

The molecular target for the classical complement pathway (CP) is defined by surface-bound immunoglobulins. Therefore, numerous anticancer monoclonal antibodies (mAbs) exploit the CP as their effector mechanism. Conversely, the alternative complement pathway (AP) is spontaneously induced on the host and microbial surfaces, but complement inhibitors on host cells prevent its downstream processing. Gain-of-function (GoF) mutations in the AP components that oppose physiological regulation directly predispose carriers to autoimmune/inflammatory diseases. Based on the homology between AP and CP components, we modified the CP component C2 so that it emulates the known pathogenic mutations in the AP component, factor B. By using tumor cell lines and patient-derived leukemic cells along with a set of clinically approved immunotherapeutics, we showed that the supplementation of serum with recombinant GoF C2 variants not only enhances the cytocidal effect of type I anti-CD20 mAbs rituximab and ofatumumab, but also lowers the threshold of mAbs necessary for the efficient lysis of tumor cells and efficiently exploits the leftovers of the drug accumulated in patients’ sera after the previous infusion. Moreover, we demonstrate that GoF C2 acts in concert with other therapeutic mAbs, such as type II anti-CD20, anti-CD22, and anti-CD38 specimens, for overcoming cancer cells resistance to complement attack.

## 1. Introduction

The introduction of complement-activating anticancer monoclonal antibodies (mAbs) as a means of targeted therapy opened a new era in oncology and hematology. The first-in-class drug, anti-CD20 rituximab approved in 1997, has markedly reduced the mortality of patients with non-Hodgkin lymphoma (NHL) [1]. Despite an undoubted success, the number of patients poorly responding or refractory to rituximab, declining efficacy of subsequent rounds of therapy, and limited efficacy in other B-cell malignancies imposed studies on new generations of anti-CD20 mAbs [2]. The second-generation anti-CD20 mAb ofatumumab presented superior in vitro complement-dependent cytotoxicity (CDC) [3]. However, data from randomized clinical studies demonstrated no superior efficacy versus rituximab [4], thus suggesting a dispensable role of complement activation in the therapeutic effect of anti-CD20 mAbs. On the other hand, a post-infusion drop in complement activity of patients’ sera together with the observation that rituximab-nonresponsive patients achieved clinical response when the infusion was accompanied with fresh frozen plasma suggest complement exhaustion as an important factor limiting the efficacy of anti-CD20 mAbs (reviewed in [5]). Moreover, tumor cells overexpress and hijack complement inhibitors, which speed up unproductive complement consumption [6,7]. Rituximab and ofatumumab are classified as type I anti-CD20 mAbs, i.e., strong activators of CDC and weak direct inducers of cell death. The opposite characteristic is a hallmark of type II specimens represented by glycoengineered, third-generation anti-CD20 mAb obinutuzumab [8]. By triggering different effector mechanisms, obinutuzumab may overcome the resistance to type I antibodies. Improvements in progression-free survival were presented in clinical trials of head-to-head comparisons of obinutuzumab and rituximab in patients with treatment-naïve chronic lymphocytic leukemia (CLL) and follicular lymphoma [9]. However, recent data from a large phase 3 trial including patients with untreated diffuse large B cell lymphoma (DLBCL) demonstrated no advantage over rituximab treatment [10]. Therefore, there is still room for improvement of existing anti-CD20 therapies and an unmet clinical need for the effective treatment of certain B-cell malignancies. Herein we tested the suitability of complement C2 variants encompassing multiple gain-of-function (GoF) mutations as supporters of mAb-based immunotherapy.

The concept is based on known GoF mutations in the alternative complement pathway (AP) that predispose carriers to autoimmune/inflammatory diseases. Nearly half of the complement system proteins act as inhibitors that disable unwanted activation on the cell surfaces [11]. Most of these inhibitors restrain complement convertases—key enzymatic complexes that augment the complement activation by catalyzing the breakdown of C3 and C5 proteins. The mechanism of convertase regulation involves either support of their proteolytic disruption or boosting their irreversible dissociation [11]. Due to the spontaneous activation of AP and a constant need for controlling it on host cells, any mutations in AP convertase components that render them insensitive to physiological regulation are potentially pathogenic. Conversely, the target for the classical complement pathway (CP) is defined by surface-bound immunoglobulins. Therefore, analogous GoF mutations in CP components would counteract the intrinsic mechanisms of tumor cells’ resistance to mAb-based cancer immunotherapeutics. Factor B and C2 proteins are components of the AP and CP convertases, respectively. They share a high degree of amino acid similarity (Figure S2), almost identical structural organization (Figure S1) [12], and perform analogous roles in their pathways by acting as enzymatic components providing a serine protease activity. Several missense mutations in factor B (e.g., p. D279G, p. Y363A, p. M458I) that result in the GoF phenotype were identified in patients with rare kidney diseases [13,14] or designed in silico [15]. We translated some of these mutations to corresponding substitutions in C2 protein (Figures S1 and S2). Then, to obtain C2 variants providing several independent modes of the classical convertase enhancement, we combined these mutations into one construct. The goal of our work was to provide evidence that such in silico designed, putative GoF mutations in C2 improve the complement-dependent cytotoxicity of anticancer monoclonal Abs.

## 2. Methods

### 2.1. Protein Expression and Purification

Wild-type C2 cDNA sequence (accession number NM_000063.5) additionally containing six histidine codons at 3′ terminus, as well as sequences for single, double, triple and quadruple variants, were codon-optimized, synthesized and cloned into pCEP4 vector in the framework of GeneArt Gene Synthesis service by Thermo Fisher (Waltham, MA, USA) as described in [16]. Plasmid DNA was transfected into HEK293 Freestyle cells (Thermo Fisher) using Freestyle Max reagent (Thermo Fisher). Conditioned FreeStyle 293 expression medium (Thermo Fisher) was collected at the 2nd, 4th and 7th days post-transfection and stored at −80 °C until the protein purification. The resulting proteins were purified with HisTrap FF affinity column (GE Healthcare) (Chicago, IL, USA), and elution was carried out with an 0.7 M imidazole gradient. Buffer was exchanged to PBS and the purity was confirmed by SDS-PAGE followed by Coomassie staining.

### 2.2. In Vitro Culture of Human Lymphoma Cell Lines and Primary CLL Cells

All cell lines (Raji, Ramos, Daudi, Namalwa, SU-DHL-4) were obtained from American Type Culture Collection (ATCC). Cells were aliquoted and cryopreserved after the first few passages. Cells used for experiments were grown from such stock aliquots and cultured in RPMI 1640 medium with L-glutamine (ATCC) supplemented with 10% fetal bovine serum (ATCC) at 37 °C and humidified 5% CO_2_ atmosphere. All cell lines were routinely checked for mycoplasma contamination by DAPI staining when cultured and never kept in a continuous culture for more than 10 passages. The cultures of primary CLL cells were established as previously [5] and cultured in a 1:1 mixture of RPMI 1640:DMEM (HyCult) medium supplemented with 20% FBS.

### 2.3. Clinical Material

Serum samples were collected from patients admitted to the Dept. of Hematology, Medical University of Gdańsk. The inclusion criterion was a diagnosis of B-cell malignancy with no prior treatment with anti-CD20 mAbs. All samples collected from patients and healthy volunteers were obtained after written informed consent, in accordance with the Declaration of Helsinki and with approval from The Local Bioethical Committee at Medical University of Gdańsk (approval number: NKBBN/500/2016). Blood collection, sample handling and storage were performed as described in Ref. [5]. Briefly, blood was collected into Vacutainer tubes with clot activator (BD Biosciences) before and after each intra-venal infusion of standard rituximab dose (375 mg per 1 m^2^ of body surface). Isolated blood was left at room temperature until clot formation, centrifuged at 700× *g* for 12 min at 4 °C, then pooled, centrifuged again to remove residual cells, aliquoted, and finally stored at −80 °C until needed. The same procedure was applied for blood collection from healthy volunteers used for the preparation of normal human serum (NHS). Heat inactivation of NHS was performed by incubation at 56 °C for 30 min following by centrifugation at 2500 RCF.

### 2.4. CDC Assay

Complement-dependent cytotoxicity (CDC) assay was performed as described in [5]. Briefly, cells were harvested (1 × 10^5^ cell per experimental point) and loaded with Calcein-AM for 30 min at 37 °C (1 mg/mL diluted in complete medium). Afterward, cells were washed twice with PBS, pelleted in the V-shaped plate, and overlaid with respective serum and sensitizing antibody solution followed by 30 min incubation with shaking (37 °C, 600 rpm). Fluorescence of collected supernatant was measured at 490/520 nm in Synergy H1 microplate reader (BioTek) (Winooski, VT, USA). Cell lysis was calculated in reference to the readout obtained for cells treated with 30% DMSO. The readout of the sample incubated with heat-inactivated serum indicated a background calcein release. Another version of the CDC assay was aimed to assess the bystander lysis of erythrocytes. For that purpose, human heparinized blood was washed twice and stored in Alsever’s solution, and then washed and diluted 1:1 in PBS buffer with calcium and magnesium. Then, 10 μL of blood solution was added to the wells containing the CD20^+^ cells and NHS +/− sensitizing antibody and/or the GoF C2 variant. To evaluate the maximal attainable release of hemoglobin, a full lysis control was established by the addition of 50 μL of water to the wells containing CD20^+^ cells and erythrocytes. After 30 min, the cells were pelleted, and the absorbance of the supernatant was measured at 405 nm.

### 2.5. Classical Convertase Assays

CP convertase assay was performed as described in [17], with further modifications described in [18]. Briefly, cells were loaded with calcein AM, then pelleted in a V-shaped plate, overlaid with 25 µL of PBS, and incubated at 37 °C with shaking at 600 rpm. A mixture of ofatumumab (100 µg/mL) and 20% of C5-depleted serum (Complement Technology) (Tyler, TX, USA) supplemented with 5 µg/mL of respective C2 variant (all in a total volume of 25 µL) were added at a specific time point. Then, cells were washed with EDTA-GVB buffer (40 mM EDTA, 5 mM veronal buffer, 0.1% gelatin, 145 mM NaCl), pelleted, and overlaid with EDTA-GVB buffer containing 1:20 dilution of guinea pig serum (Harlan Laboratories) (Indianapolis, IN, USA). The readout of calcein release was performed as in the CDC assay.

### 2.6. Flow Cytometry

The expression of CD20, CD46, CD55 and CD59 was analyzed, employing flow cytometry, as noted in [19]. Primary antibodies (clone UJ11 for CD35, clone MEM-258 for CD46, and clone HI-55a for CD55; all from Immunotools) and rituximab were used, followed by detection with secondary goat-anti mouse F(ab)2 labelled with AF488 antibody (Dako, 1:200 dilution). The deposition of C4b and C3b was analyzed using polyclonal goat anti-human C4 antibody (Complement Technology, 1:200 dilution) or rabbit anti-human C3c-FITC (Dako, 1:200 dilution), respectively. Non-conjugated primary anti-C4 antibody was detected by donkey anti-goat Alexa Fluor488 antibody (Invitrogen) (Waltham, MA, USA), diluted 1:400.

### 2.7. Assessment of Rituximab Concentration

Rituximab concentration in serum samples collected just before and just after each infusion was measured using an enzyme-linked immunosorbent assay, exactly as described previously [5].

### 2.8. CD20 siRNA-Mediated Knock-Down

SU-DHL4 cells (0.5 × 10^6^/mL per well) were seeded onto a 6-well plate the day prior to transfection. Cells were transfected with CD20 siRNA duplexes (ON-TARGETplus Human MS4A1 siRNA, Dharmacon/Thermo Scientific) or siRNA negative control (Sigma) (St Louis, MI, USA) using FuGENE^®^ 6 Transfection Reagent (Promega) (Madison, WI, USA). After 48 h, the cells were subjected to a second transfection using the same experimental conditions. Forty-eight hours after re-transfection, the cells were harvested and analyzed by flow cytometry for CD20, CD46, CD55 and CD59 expression, then subjected to CDC assay.

### 2.9. Statistical Analysis

Statistical analyses were performed with GraphPad Prism 8 software. The Kruskal–Wallis test was applied for column data, while the Dunnett multiple comparison test for non-repeated measures was applied for grouped analysis.

## 3. Results

**Figure 1:**
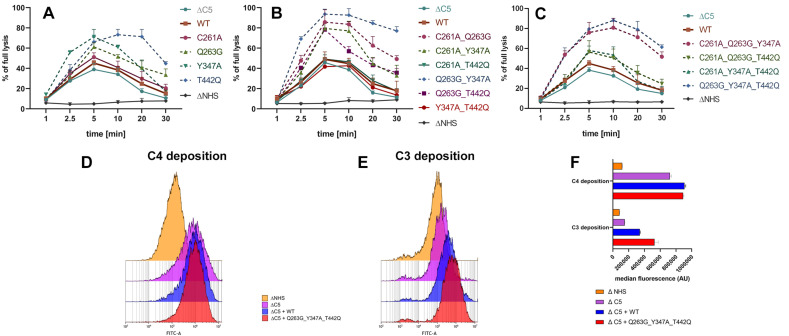
The classical pathway convertase activity. (**A**–**C**) Ramos cells were loaded with calcein-AM, sensitized with 50 μg/mL ofatumumab and incubated for the indicated time with 20% of C5-depleted serum (ΔC5) supplemented with a particular single (**A**), double (**B**) and triple (**C**) C2 mutants. Heat-inactivated serum (ΔNHS) was applied as a negative control. Then, cells were washed and suspended in guinea pig serum diluted 1:20 in EDTA-containing veronal buffer to execute lysis. Fluorescence of released calcein was measured at 490/520 nm, as described in Ref. [18]. Graphs present data from three independent experiments, and error bars show standard deviation. (**D**–**F**) Raji cells sensitized with 50 μg/mL rituximab were incubated with 5% ΔNHS (negative control) or with 5% ΔC5 supplemented with wild-type (WT) or triple (Q263G_Y347A_T442Q) GoF C2 mutant. Calculations of the statistical significance for each variant vs. WT are available in Table S1. The deposition of C4 (**D**) and C3 (**E**) was analyzed by flow cytometry to confirm the effect on GoF mutant on increased C3b but not C4b production, where the first is indicative for the CP activity. Panel (**F**) shows changes in the median fluorescence intensity recorded in three technical replicates of the flow cytometry experiment.

**Figure 2:**
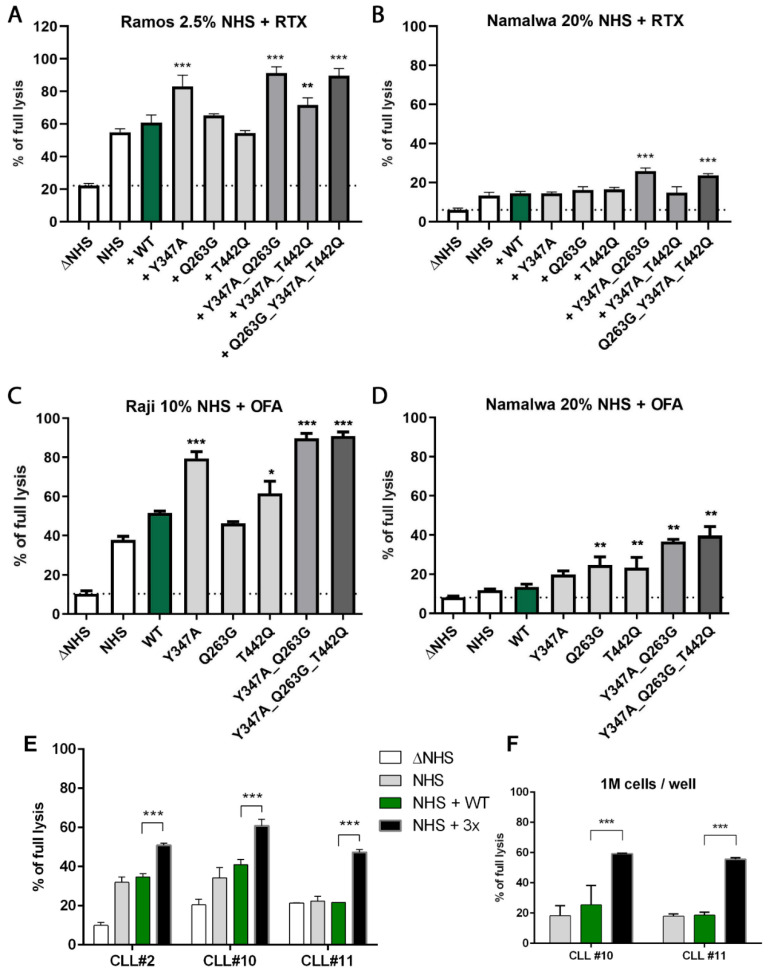
GoF C2 variants support efficient CDC of anti-CD20 therapeutic antibodies. CDC assay was performed in a way that calcein AM-labelled cells were sensitized with 50 μg/mL type I anti-CD20 antibody (ofatumumab or rituximab) and incubated for 30 min at 37 °C with indicated concentration of normal human serum (NHS) supplemented with a physiological concentration of particular C2 mutant. (**A**) CDC exerted in Ramos cell line sensitized with rituximab, (**B**) CDC exerted in rituximab-sensitized Namalwa cells, (**C**) CDC exerted in ofatumumab-sensitized Raji cells, (**D**) CDC exerted in ofatumumab-sensitized Namalwa cells. The green bar indicates supplementation with wild-type (WT) protein. (**E**,**F**) Primary CLL cells were employed for CDC assay in 50% NHS supplemented with physiological concentration of wild type (WT) triple GoF C2 variant (3×). All experiments shown in panels (**A**–**E**) were performed with 10^5^ cells per well, whereas experiment in panel (**F**) was performed with 10^6^ cells per well. Heat-inactivated serum (ΔNHS) was considered a background, complement independent lysis and depicted by a dotted line. Statistical significance at *p* level < 0.05 *, *p* < 0.01 ** and *p*  <  0.001 *** in comparison to cell lysis obtained by the addition of wild-type C2 (WT, green bar) was calculated according to Dunnett’s multiple comparison test (GraphPad Prism). Graphs present data from three independent experiments, and error bars show standard deviation.

First, we verified the effect of single and multiple substitutions in C2 on the activity of CP convertase formed on the surface of CD20-positive human lymphoma cells sensitized with ofatumumab. Our results confirmed the elevated activity of CP convertases assembled from some of the designed C2 variants carrying single (Figure 1A), double (Figure 1B) or triple (Figure 1C) predicted GoF mutations. To additionally confirm that these variants act at the stage of complement convertases, we analyzed the deposition of C4 and C3 proteins on the cell surface. Supplementation of serum with triple (Q263G_Y347A_T442Q) GoF mutant chosen for this experiment revealed no differences in C4 deposition but enhanced C3 deposition compared to supplementation with wild-type C2 (Figure 1D–F). Then, we tested best-performing single and multiple GoF mutants in CDC assays on three cell lines of high (Ramos), moderate (Raji), and low (Namalwa) sensitivity to anti-CD20-mediated CDC, a derivative of the net effect of either quantity and composition of complement inhibitors or the expression of CD20 on the surface [19]. Importantly, a 10-fold increase in therapeutic antibodies rituximab or ofatumumab from 50 to 500 μg/mL did not significantly increase CDC in tested cell lines (Figure S3), unlike the supplementation of serum with certain GoF mutants (Figure 2A–D). We repeated the same experiments with chosen C2 variant encompassing three single GoF mutations (Q263G, Y347A, and T442Q, subsequently called “triple” mutant) on fresh cultures of CLL cells isolated from three treatment-naïve patients. CLL presents a major clinical challenge for anti-CD20 therapy, as these cells express low levels of target antigen compared to non-Hodgkin’s lymphomas [19]. The triple GoF mutant significantly increased CDC exerted by ofatumumab in each CLL culture (Figure 2E). The same result was observed when cell density increased ten-fold (Figure 2F), thus suggesting that GoF C2 mutant can be effective at a high tumor burden.

**Figure 3:**
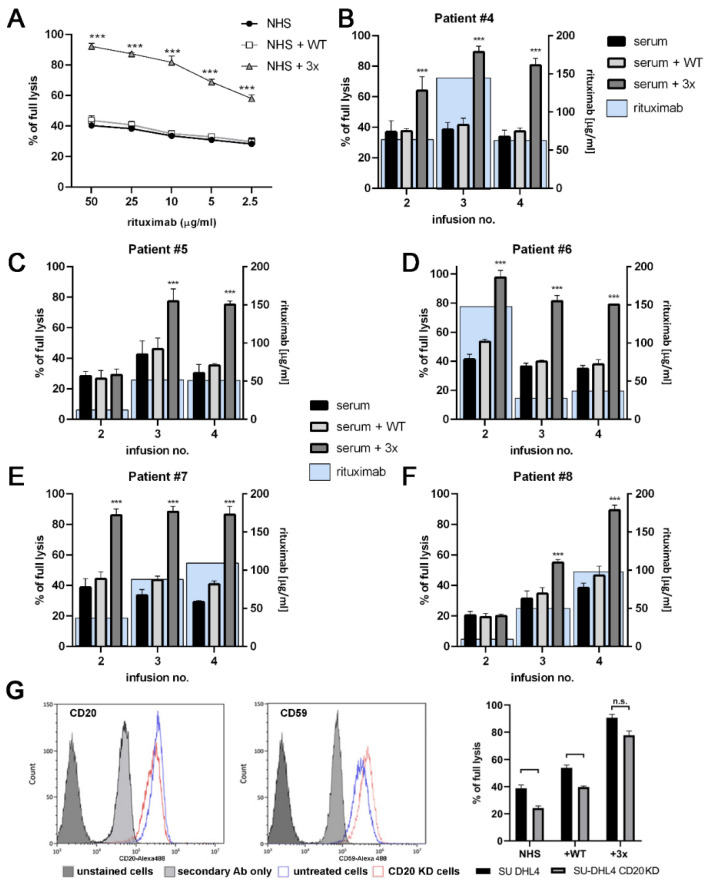
Demonstration of C2 triple GoF mutant capacity to support CDC in suboptimal conditions. Panel (**A**) shows CDC mediated by rituximab on Raji cells upon a decreasing concentration of the drug after serum supplementation with wild-type (WT) or triple (3x) GoF C2 variant. Panels (**B**–**F**) present the effect of supplementation of patients’ sera collected before the 2nd, 3rd and 4th infusions of rituximab on CDC. Blue rectangles indicate the concentration of rituximab analyzed by ELISA, as described in Ref. [3]. Symbol *** depicts statistical significance of differences between the results for WT and 3x supplementation at *p* < 0.001, according to Dunnett’s multiple comparison test. (**G**) The left panels show histograms comparing CD20 and CD59 levels on the surface of SUDHL-4 line with CD20 siRNA-mediated knockdown (CD20KD) or untreated cells. Dark grey peaks indicate unstained cells, while light grey peaks mark cells stained with secondary Ab only. Red and blue peaks present the level of CD20 (left histogram) or CD59 (right histogram) expression for either SUDHL control or CD20 knockdown cells. These cells were then used in CDC assay testing the effect of supplementation with WT or 3X variants of C2 protein (chart on the right). *** refers to statistical significance at *p* < 0.001 between CD20KD and untreated cells, according to Dunnett’s multiple comparison test.

Afterward, we tested whether the triple GoF C2 mutant can trigger efficient CDC at low rituximab concentrations. Raji cells were treated with decreasing concentrations of rituximab, and the addition of triple mutant supported CDC with double the effectiveness as the addition of wild-type C2. This effect occurred within the whole concentration range of rituximab, from 50 to 2.5 μg/mL. Even at the lowest rituximab concentration, the CDC activity of GoF mutant-containing serum was markedly higher than CDC at the highest rituximab concentration in non-supplemented or wild-type C2-supplemented serum (Figure 3A). To further exploit the potential of the triple C2 variant to maximize CDC at a limited concentration of anti-CD20 mAbs, we examined sera from five NHL patients treated with rituximab, which were collected before the second, third, and fourth infusions of the drug administered in four-week intervals. The only source of rituximab present in these samples was accumulation after the previous infusions. The results showed that upon supplementation of patients’ sera with triple GoF C2 mutant, the leftovers of rituximab exerted substantial CDC in most of the cases (Figure 3B–F), thus confirming that hyperactive classical convertases significantly decrease the threshold of mAb concentration necessary for the efficient killing of tumor cells by host immune effectors.

To approach the possible problem of off-target lysis in the presence of the GoF C2 variants, we performed a CDC assay with no sensitizing antibodies added to the test tube. No elevated CDC was observed when serum was supplemented with triple GoF C2 mutant (Figure S4A). We also did not observe significant bystander lysis of erythrocytes co-incubated with antibody-sensitized Raji cells and hyperactive C2 (Figure S4B).

The resistance of CD20-positive cells to CDC is a derivative of a target antigen to complement inhibitors ratio, as shown in [19]. Takei et al. tracked the process of gaining resistance to rituximab by Ramos cells, which concurrently diminished CD20 expression and overexpressed complement inhibitors [20]. Using the siRNA technique aimed to knock down CD20 expression, we succeeded in isolating the SU-DHL-4 cells with a decreased cell-surface level of target antigen and also increased level of membrane-bound complement inhibitor CD59 (Figure 3G). However, we observed much higher readout obtained for CD20^low^/CD59^high^ cells in serum supplemented with triple GoF C2 variant than for parental CD20^high^/CD59^low^ cells in non-supplemented or wild-type C2-supplemented serum (Figure 3G). The conclusion is that multiple GoF C2 mutant not only ensures maximal CDC efficacy of rituximab, but also counterbalances the selection of resistant cells.

**Figure 4:**
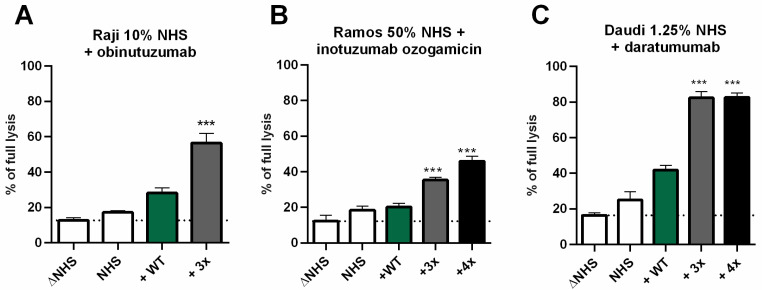
Triple GoF C2 variant induces CDC on cells sensitized with poor complement activators. Raji, Ramos or Daudi cells were incubated with normal human serum (NHS) at indicated concentration, supplemented with a physiological concentration of wild-type (WT), triple (3x), or quadruple (4x) C2 variant and indicated antibody to induce CDC. (**A**) Raji cells were sensitized with type II anti-CD20 mAb obinutuzumab. (**B**) Ramos cells were incubated with anti-CD22 (inotuzumab ozogamicin). (**C**) Daudi cells were sensitized with anti-CD38 antibody (daratumumab). Heat-inactivated serum (ΔNHS) was considered as a background and complement-independent lysis and is depicted by a dotted line. Statistical significance at *p* level < 0.001 *** in comparison to cell lysis obtained by the addition of wild-type C2 (WT, green bar) was calculated according to Dunnett’s multiple comparison test (GraphPad Prism). Graphs present data from three independent experiments and error bars show standard deviation.

To examine if the enhancement of CDC by GoF C2 variants extends to anticancer antibodies other than type I anti-CD20 mAbs, we performed assays with anti-CD38 mAb daratumumab and two antibodies reported to poorly activate the complement: obinutuzumab (a type II anti-CD20 mAb), and anti-CD22 mAb inotuzumab ozogamicin. All antibodies supplemented with triple C2 mutant exerted significantly higher CDC compared to supplementation with wild-type C2 (Figure 4). In some of these experiments, we also tested the quadruple mutant (a triple mutant with additional C261A substitution—see Figure S1 for more explanations), which showed higher CDC than triple one when used with inotuzumab ozogamicin (Figure 4B).

## 4. Discussion

The effective therapeutic dose of type I anti-CD20 mAbs is a matter of ongoing debate. On one hand, increasing the standard rituximab dose (375 mg/m^2^) was proposed in individuals with a high tumor burden that affects the pharmacokinetics of the drug and can lead to suboptimal exposure [21]. On the other hand, Kennedy et al. reported that application of the standard rituximab dose can deplete the complement in CLL patients for a week or longer [22]. Beurskens et al. extended these findings and showed that at a physiologically relevant range of tumor cell burden, application of an excessive amount of type I mAbs can severely deplete complement activity in 50% serum [23]. In their two-step experiments, which mimicked repetitive dosing of mAb, large amounts of antibodies deposited C3 on the surface of CLL cells; however, a saturation of CDC was achieved at much lower antibody concentration than the saturation of C3 deposition. Application of the amount of antibody exceeding the CDC saturation level resulted in the exhaustion of complement activity and severely compromised CDC upon challenge with additional tumor cells, even if adequate mAb concentrations were present. Another important observation made in rituximab-receiving CLL patients was that up to 80% of circulating tumor cells were eliminated after infusion of as little as 30 mg of mAb, but the leukemic cells count rebounded shortly after completing the infusion with the remaining dose [24]. Notably, these repopulating cells had markedly (90–95%) reduced levels of CD20 on their surface, which could stem from trogocytosis initiated by excessively administrated mAbs [24,25]. All the above-mentioned remarks indicate the necessity to maximize the cytocidal effect from the single anti-CD20 mAb particle. Supplementation of serum with triple GoF C2 mutant fulfills such a need and leads to the reduction in the effective antibody dose, as shown in Figure 3. Another advantage of GoF C2 mutant was potentiation of CDC exerted by type I anti-CD20 mAbs toward resistant CLL cells (Figure 2E,F) as well as an ability to release substantial CDC from antibodies characterized by a weak potential of complement activation (Figure 4). Recently, Kumar et al. provided a model of binding of anti-CD20 therapeutics to its target [26]. The results revealed that type I mAb forms 1:2 or 2:1 (mAb:CD20) “seeding” complexes that enable subsequent concatenation of mAb or CD20 molecules, respectively. Conversely, obinutuzumab forms 1:2 “terminal” complexes that preclude the binding of additional mAb molecules, thus preventing the hexamerization required for efficient C1q recruitment. Therefore, obinutuzumab would require higher antigen densities for oligomerization and complement activation. This model explains the difference between the ability of type I and II anti-CD20 antibodies to activate CDC. However, our experiments show that despite suboptimal binding mode to CD20, obinutuzumab may exert significant CDC when supplemented with triple GoF C2 mutant (Figure 4A). One may speculate that at a favorable local density of CD20, even minimal complement activation could be sufficient for effective CDC when the classical convertase is formed by hyperactive C2 protein. Inotuzumab ozogamicin is a humanized IgG4 anti-CD22 mAb bound covalently to calicheamicin dimethyl hydrazide that mediates apoptosis in target cells [27]. Notably, the IgG4 isotype poorly induces Fc-dependent effector functions including CDC due to specific conformation of the Cγ2 domain [28]. However, triple and quadruple GoF C2 variants but not wild-type C2 added to 50% human serum exerted substantial lysis of Ramos cells (Figure 4B).

The proposed strategy of using hyperactive C2 variants has several advantages over other attempts to overcome the resistance of tumor cells to CDC. Neutralization of complement inhibitors as a concept to increase the efficacy of therapeutic antibodies was exploited by testing bi-specific antibodies targeting CD20 and CD55 either in vitro or in a murine xenograft model of Burkitt lymphoma [29]. Additionally, the siRNA-mediated silencing of membrane complement inhibitors was proposed [30]. However, the affinity of the bi-specific antibody is limited to only one type of inhibitor and does not neutralize the activity of other inhibitors, e.g., factor H, which is present in serum in micromolar concentration and, when hijacked by tumor cells, contributes to anti-CD20 mAb resistance [6]. Silencing of complement inhibitors by siRNA may be problematic due to the expression of target molecules on host cells, thus requiring a specific delivery of siRNA only to tumor cells. The abovementioned limitations are not valid in the case of hyperactive C2 variants that generally oppose the activity of complement inhibitors and become activated in response to antibodies.

Although our concept assumes only temporal supplementation of C2 GoF variants that is strictly coordinated with the administering of anticancer antibodies that define a molecular target, we believe that potential safety issues must be addressed in a separate preclinical study in a valid in vivo model. Nonetheless, there are premises that the concept is clinically applicable. We did not observe the off-target effects of the GoF C2 variant and bystander lysis of erythrocytes co-incubated with antibody-challenged lymphoma cells (Figure S4). Hyperactive convertases of the alternative complement pathway are potentially detrimental, as AP spontaneously deposits C3b on the host’s cells and tissues. Indeed, patients with GoF mutations in AP convertase components often develop renal diseases at a very young age [31]. Recently, we identified patients that carry natural GoF mutations in the C2 gene. They developed glomerulopathies at the age >50 and had no history of other diseases, despite the presence of anti-nuclear autoantibodies and risk haplotypes in other complement genes (MCP, CFH) [32]. This suggests that the existence of hyperactive CP convertases does not automatically result in organ and tissue damage and that it takes dozens of years of the continuous presence of the GoF C2 variant to develop a specific pathology.

## 5. Conclusions

Our data confirmed the ability of GoF C2 variants to boost either the cytocidal activity of antibodies normally not engaged in complement activation or maximize the cytotoxic yield from a limited amount of type I anti-CD20 mAbs. The low physiological serum concentration of C2 protein (25 μg/mL) compared to other constituents of alternative and classical convertases makes it a bottleneck of the whole classical pathway and offers a possibility of effective supplementation with relatively low amounts of recombinant protein. A clear advantage of the herein proposed strategy is the compatibility of GoF C2 with numerous therapeutic antibodies and the possibility to combine different mutations that oppose the activity of several complement inhibitors into a single construct.

## Data Availability

Data are contained within the article and Supplementary Material. The raw data are available on request from the corresponding author.

## Supplementary Materials

The following supporting information can be downloaded at: https://www.mdpi.com/article/10.3390/cancers14051270/s1. Figure S1 is a graphical presentation of known GoF mutations in complement factor B and corresponding mutations translated into complement C2 structure. Figure S2 shows alignment of the amino acid sequences of von Willebrand factor A domains of C2 and FB. Figure S3 shows that increasing in the concentration of therapeutic antibodies does not lead to significantly increased CDC in human CD20+ cell lines. Figure S4 presents in vitro experiments on off-target effects of the GoF C2 variant. Table S1 presents the statistical calculations related to the results shown in Figure 1.

## Abbreviations

ATCC—American Type Culture Collection; AP—alternative complement pathway; CDC—complement-dependent cytotoxicity; CLL—chronic lymphocytic leukemia; CP—classical complement pathway; GoF—gain-of-function; mAb—monoclonal antibody; NHL—non-Hodgkin lymphoma
