# Supplementary material for: In Silico Designed Gain-of-Function Variants of Complement C2 Support Cytocidal Activity of Anticancer Monoclonal Antibodies
Source: Cancers (Basel). 2022 Mar 1;14(5):1270. doi: 10.3390/cancers14051270 (PMC8909654; doi:10.3390/cancers14051270)
Supplement: Supplementary file 1 [file cancers-14-01270-s001.zip › cancers-1600347-supplementary.pdf]

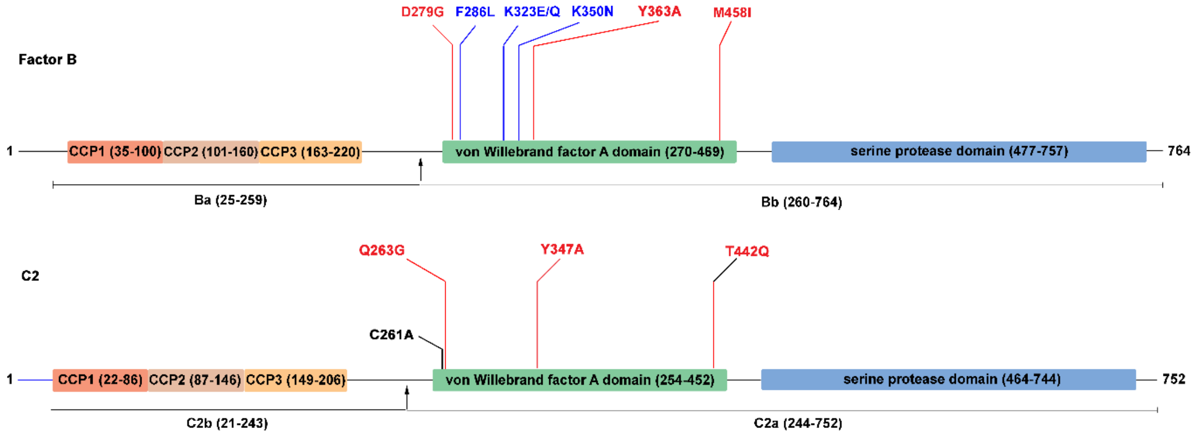

Figure S1. Homology of Factor B and complement C2 proteins. The scheme presents the placement of domains within the primary structure of paralogs: factor B (upper graph) and C2 (bottom graph). Black arrows mark cleavage sites in factor B and C2. Gain-of-function mutations in factor B confirmed with functional studies are indicated and mutations chosen for translation into the corresponding substitutions in C2 are marked with red font. Mutation C261A indicated in black font, which was not reported in factor B but claimed to improve the intrinsic stability of classical convertase (*Mortensen et al., J Biol Chem. 2016, 291: 16494-507*) was also included.

```
C2 251   GHLNLYLLLDCSQSVSENDFLIFKESASLMVDRIFSFEINVSVAIITFASEPKVLMSVLN   310
         G +N+YL+LD S S+  ++F   K+    +++++ S+ +     ++T+A+ PK+ + V
FB 267   GSMNIYLVLDGSDSIGASNFTGAKKCLVNLIEKVASYGVKPRYGLVTYATYPKIWVKVSE   326

C2 311   DNSRDMTEVISSLENANYKDHENGTGTNTYAALNSVYLMMNNQMRLLGMETMAWQEIRHA   370
          +S +   V   L   NY+DH+  +GTNT  AL +VY MM+       +    W   RH
FB 327   ADSSNADWVTKQLNEINYEDHKLKSGTNTKKALQAVYSMMSWPD---DVPPEGWNRTRHV   383

C2 371   IILLTDGKSNMGGSPKTAVDHIREILNINQKRN----DYLDIYAIGVGKLDVDWRELNEL   426
         IIL+TDG  NMGG P T +D IR++L I + R     DYLD+Y  GVG L V+   +N L
FB 384   IILMTDGLHNMGGDPITVIDEIRDLLYIGKDRKNPREDYLDVYVFGVGPL-VNQVNINAL   442

C2 427   GSKKDGERHAFILQDTKALHQVFEHMLDVSKLTDTICGVGNMSANASDQERTPWHV---T   483
          SKKD E+H F ++D + L  VF  M+D S+ + ++CG+       +D  + PW
FB 443   ASKKDNEQHVFKVKDMENLEDVFYQMIDESQ-SLSLCGMVWEHRKGTDYHKQPWQAKISV   501
```

Figure S2. Alignment of the amino acid sequences of von Willebrand factor A domains of C2 (upper) and FB (bottom). Using the same color code as in Fig. S1, the alignment shows the amino acids, which substitution results in the GoF phenotype in FB. Three of these substitutions at positions indicated by red boxes were translated from FB to C2. The common amino acid letter in the middle line indicates amino acid identity, and "+" indicates similarity due to physicochemical properties. Original GoF mutation in FB at position 279 changes acidic amino acid with a spacious side chain (Asp) into small Gly. Therefore, corresponding Gln residue in C2 was also converted to Gly. Despite amino acid identity at position 286 (FB), the GoF variant with Leu instead of Phe turned out not to be possible to express in our eukaryotic system. We reported a similar problem previously (*Felberg at al., Cancer Immunol Immunother. 2019; 68: 587-598*). There was no amino acid in the C2 sequence that functionally corresponds to Lys at position 323 in FB. We expressed C2 variant p.S307E (as K->E substitution produces GoF variant of FB), but it did not show the GoF phenotype (not shown). The region surrounding position 350 in FB was different in C2, so we abandoned the construction of the homologous C2 variant. Substitution of Tyr to Ala at position 363 in FB leading to GoF phenotype could be reproduced in C2. The region around residue 458 in FB is conserved in C2 but the residue 442, (hydrophobic Met in FB, polar Thr in C2), and the originally reported GoF phenotype in FB embraced substitution of one hydrophobic to another hydrophobic residue. Therefore, we examined a C2 construct with substituted polar, uncharged residue (Thr) to the same category of the amino acid (Gln).

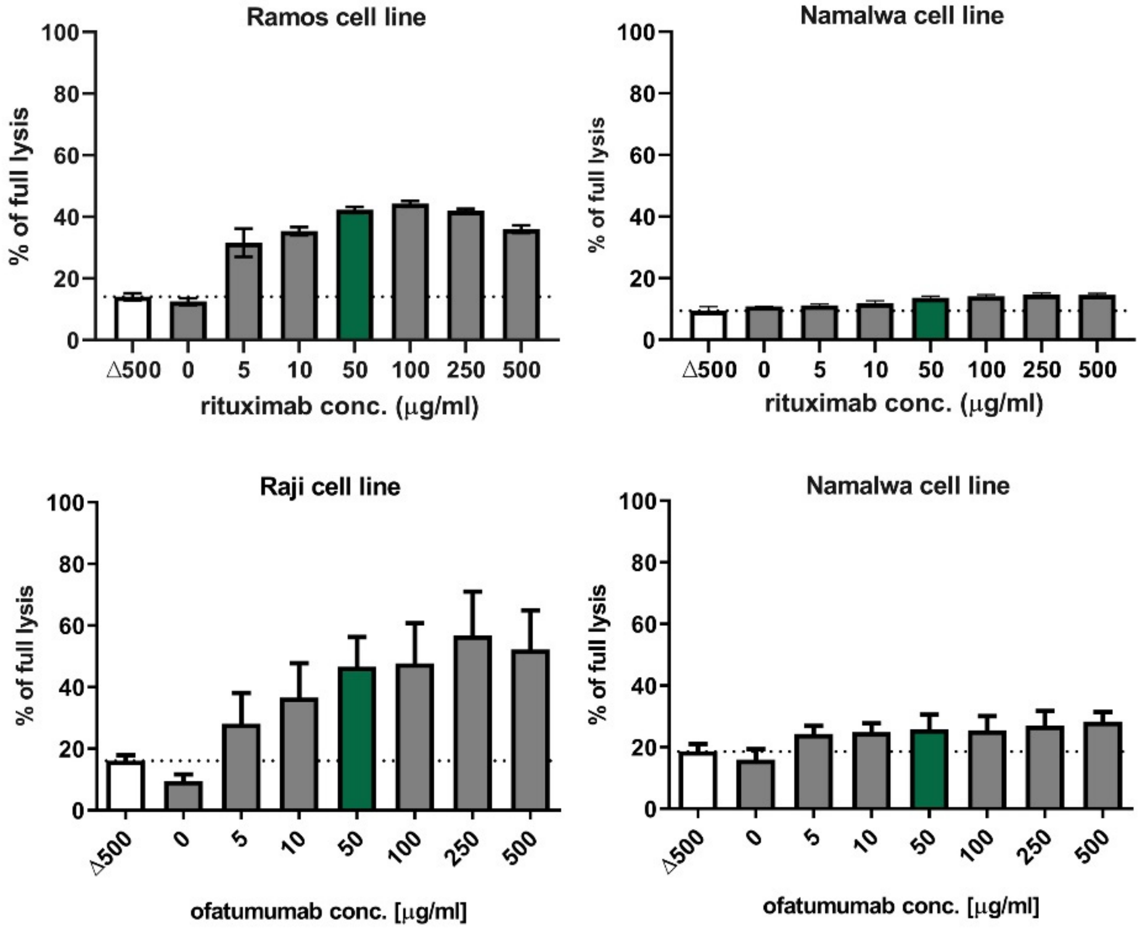

Figure S3. Calcein-AM-labelled Ramos, Raji, or Namalwa cells were sensitized with anti-CD20 antibody (ofatumumab or rituximab). The graphs present the CDC of each cell line after titration of a particular antibody. The green bar indicates saturating concentration of antibodies (50 μg/ml), which was further applied in all experiments. Heat-inactivated serum supplemented with the highest concentration of the drug (Δ500) was considered as a background and is depicted by a dotted line. Graphs present data from three independent experiments and error bars show standard deviation.

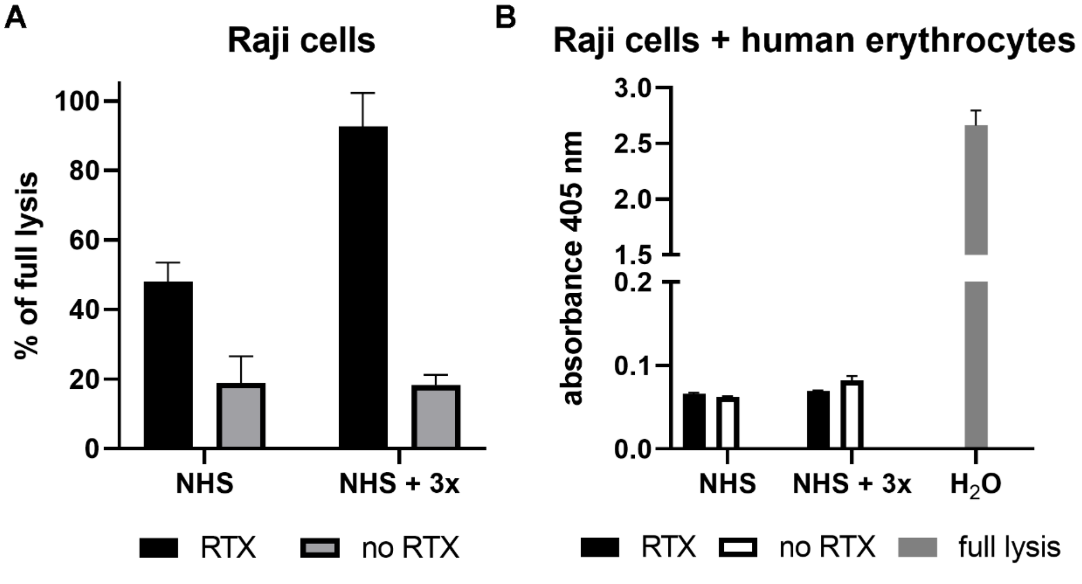

Figure S4. Assessment of off-target lysis in the presence of triple C2 GoF mutant. (A) The CDC assay was performed on Raji cells incubated with 10% NHS and 50 μg/ml rituximab. Alternatively, cells

were incubated with NHS only (without rituximab), with NHS supplemented with 3x C2 variant and rituximab, or with the same composition of NHS and 3x C2 but without rituximab. The assay was conducted and developed as described in the Methods section. In another version of the above experiment (B), human heparinized blood was added to the wells containing the Raji cells suspended in the same solutions as in (A). To evaluate the maximal attainable release of hemoglobin, a full lysis control was established by the addition of 50 µl of water to the wells containing Raji cells and erythrocytes. After 30 min cells were pelleted, and the absorbance of the supernatant was measured at 405 nm.

Table S1. Statistical significance of convertase activity assay readouts in comparison to cell lysis obtained by the addition of wild type C2 (WT) in Fig.1. The asterisk symbols *, **, and *** indicate p level < 0.05, p < 0.01, and p < 0.0001, respectively, according to Dunnett's multiple comparison test (GraphPad Prism).

**significance vs. wild-type C2:**	**1 min**	**2.5 min**	**5 min**	**10 min**	**20 min**	**30 min**
C261A	-	-	-	-	-	-
Q263G	-	-	**	**	**	***
Y347A	-	***	***	***	***	-
T442Q	-	-	***	***	***	***
	1 min	2.5 min	5 min	10 min	20 min	30 min
C261A_Y347A	-	-	***	***	***	**
C261A_Q263G	-	***	***	***	***	***
C261A_T442Q	-	-	-	-	-	-
Q263G_Y347A	-	***	***	***	***	***
Q263G_T442Q	-	**	***	*	***	***
Y347A_T442Q	-	-	-	-	-	-
	1 min	2.5 min	5 min	10 min	20 min	30 min
C261A_Q263G_Y347A	-	***	***	***	***	***
C261A_Q263G_T442Q	-	-	*	*	-	-
C261A_Y347A_T442Q	-	-	*	**	-	-
Q263G_Y347A_T442Q	-	***	***	***	***	***
